# Plant Products Affect Growth and Digestive Efficiency of Cultured Florida Pompano (*Trachinotus carolinus*) Fed Compounded Diets

**DOI:** 10.1371/journal.pone.0034981

**Published:** 2012-04-20

**Authors:** Gregory P. Lech, Robert C. Reigh

**Affiliations:** Aquaculture Research Station, Louisiana State University Agricultural Center, Baton Rouge, Louisiana, United States of America; Laboratoire Arago, France

## Abstract

Costs of compounded diets containing fish meal as a primary protein source can be expected to rise as fish meal prices increase in response to static supply and growing demand. Alternatives to fish meal are needed to reduce production costs in many aquaculture enterprises. Some plant proteins are potential replacements for fish meal because of their amino acid composition, lower cost and wide availability. In this study, we measured utilization of soybean meal (SBM) and soy protein concentrate (SPC) by Florida pompano fed compounded diets, to determine the efficacy of these products as fish meal replacements. We also calculated apparent digestibility coefficients (ADCs) for canola meal (CM), corn gluten meal (CGM), and distillers dried grains with solubles (DDGS), following typical methods for digestibility trials. Juvenile Florida pompano were fed fish-meal-free diets containing graded levels of SBM and SPC, and weight gain was compared to a control diet that contained SBM, SPC, and fish meal. Fish fed diets that contained 25–30 percent SBM in combination with 43–39 percent SPC had weight gain equivalent to fish fed the control diet with fish meal, while weight gain of fish fed other soy combinations was significantly less than that of the control group. Apparent crude protein digestibility of CGM was significantly higher than that of DDGS but not significantly different from CM. Apparent energy digestibility of DDGS was significantly lower than CGM but significantly higher than CM. Findings suggested that composition of the reference diet used in a digestibility trial affects the values of calculated ADCs, in addition to the chemical and physical attributes of the test ingredient.

## Introduction

Aquaculture production of marine finfish is expected to continue to increase to meet the world's growing demand for seafood. Many types of marine finfish aquaculture use compounded diets that contain high concentrations of protein, which is often provided by fish meal derived from wild fisheries or by animal processing by-products obtained from the commercial fishing and livestock production industries. Currently, about 60 percent of the world supply of fish meal is used in aquatic animal feeds [1]. Fish meal is an optimal protein source (i.e., supplement) for fish feeds because of its nutritional value and high palatability to fish [2]. Fish meal contains high levels of dietary essential amino acids and essential fatty acids (omega-6 and omega-3 highly unsaturated fatty acids) that promote rapid growth. However, fish meal is a finite resource that has steadily increased in price in recent years and will continue to become increasingly expensive relative to other protein supplements in the ingredient market. Rising fish meal prices are driving efforts worldwide to identify economical alternatives to fish meal in marine fish diets. The reduction, or elimination, of fish meal from compounded diets can be expected to provide economic and environmental benefits by reducing feed costs for fish producers, while lessening fishing pressure on species harvested for fish meal production, many of which also serve as important resources in the marine food web.

Certain plant-based protein supplements are of interest as fish meal replacements because of their relatively low cost and widespread availability. Soybean is one of the most promising plant-based substitutes for fish meal because of its excellent amino acid composition, which provides the best dietary essential amino acid profile among commonly available plant products in the ingredient market. Among the soybean products available for use in compounded fish feeds is high-protein, de-hulled, solvent-extracted soybean meal produced by heat-treatment and oil-extraction of full-fat soybeans. High-protein soybean meal contains about 49 percent crude protein, which is more than three-quarters of the amount of protein in commonly available fish meals, and prices of high-protein soybean meal have been about one-third the price of fish meal in recent years [3]. Thus soybean meal is an affordable and readily available protein source for fish feeds [4].

Despite the desirable protein content of some plant products, plants often contain high levels of carbohydrates which many fishes do not digest effectively. Poorly digested carbohydrates in soybeans include sucrose, and the oligosaccharides raffinose and stachyose. The presence of these carbohydrates in fish diets can cause diarrhea and reduced nutrient absorption. Carbohydrates in soybeans also can alter populations of micro-flora in the gastrointestinal tract of fishes, sometimes with negative effects [5]. Further processing of soybean meal into soybean protein concentrate removes soluble carbohydrates, which eliminates or greatly reduces many of the components that can negatively affect digestion of soybean by fishes, and increases the concentration of dietary essential amino acids per gram of product. Soybean protein concentrate contains about 65 percent crude protein, which is about the same amount of protein as commonly available fish meal. This has led to interest in soybean protein concentrate as a protein supplement for fish feeds and a potential fish meal replacement.

Soybean meal and soybean protein concentrate have been used effectively to replace a portion of fish meal in the diets of various fishes [6]–[17]. However, wider use of soybean-based diets has been hindered by negative effects associated with high soybean inclusion levels. High levels of soybean products in compounded diets have caused reduced feed intake, decreased growth, poor nutrient utilization and digestibility, negative physiological effects, and histological abnormalities among some fishes [5], [18]. Soybean meal, and to a lesser extent soybean protein concentrate, is deficient in the dietary essential amino acids lysine and methionine, and possesses compounds — e.g., proteinase inhibitors, lectins and saponins — that can affect digestion. Proteinase inhibitors can decrease protein digestibility by binding enzymes that hydrolyze protein in the gastrointestinal tract; lectins can produce histological abnormalities in cells of the gastrointestinal tract, reduce nutrient uptake, and affect enzyme activity; and saponins can affect mucosal-cell membrane permeability, decrease active uptake of nutrients, and facilitate the development of enteritis in some fishes [2]. The effects of consuming soybean meal vary in type and magnitude among fish species, but marine fishes with carnivorous feeding habits appear to be negatively affected when soybean meal constitutes a large part of the diet. Thus, establishing suitable levels of soybean products for marine finfish diets is of practical interest when soybean protein is used as a replacement for fish-meal protein.

The demand for soybean is expected to rise worldwide as this highly versatile oilseed is used increasingly in human foods, industrial products, and animal feeds. As soybean prices fluctuate with supply and demand, other plant-protein supplements could become useful in diet formulations when market conditions warrant a reduction in soybean content as a feed-cost control strategy. Among these ingredients are canola meal, corn gluten meal, and distillers dried grains with solubles. These ingredients have potential as partial replacements for soybean products, based on their nutrient content, price, and availability in the marketplace.

Canola is a genetically selected variety of rapeseed produced primarily for its oil. Canola oil is used in human foods and can be used as a replacement for fish oil in aquatic animal diets due to its favorable fatty acid profile. Canola meal is a by-product of canola oil production. As a result of the high demand for canola oil, canola meal is an affordable ingredient for fish diets. It contains 35–45 percent crude protein and varying amounts of lipid depending on the oil-extraction method used. Canola meal has an excellent dietary essential amino acid profile relative to fish meal [19] and its price is usually less than the price of soybean meal [3]. It has been used as a protein replacement for fish meal with varying results [20], [21], [22]. Maximum inclusion levels of canola meal in fish feeds are influenced by the presence of carbohydrates, tannins, sinapine (an alkaloid), and glucosinolates, which can depress feed intake, decrease nutrient availability, and disrupt digestion [19] of fishes and other monogastric animals [23].

Corn is one of the most cultivated plants in the United States. It has many food and industrial uses, and is the source of numerous by-products. One of these, corn gluten meal, is obtained when starch is extracted from corn grain and the protein portion of the grain (gluten) is separated from the remaining mash. Corn gluten meal contains about 60 percent crude protein; has a good dietary essential amino acid profile, except for deficiencies of lysine and arginine; and does not contain compounds that negatively affect digestion. However, it is usually priced higher than soybean meal or canola meal [3], which is likely to limit its use. Corn gluten meal has potential as a fish meal replacement and is often incorporated in some commercial diets [24].

Distillers dried grains is a by-product of ethanol production. It can be produced from a variety of grains, but most distillers dried grains produced in the USA is a by-product of the yeast fermentation of corn grain. To be classified as distillers dried grains with solubles, at least 75 percent of the solids present in the stillage (i.e., the mash remaining after the removal of ethyl alcohol by distillation) must be retained [25]. Distillers dried grains with solubles is available at reasonable cost due to increased ethanol production for motor vehicle fuel. Crude protein content of distillers dried grains with solubles is approximately 27 percent. It contains relatively low levels of the dietary essential amino acids lysine and methionine, and 35–40 percent carbohydrate. Despite these shortcomings, it has shown promise as a protein and energy source for some fishes [26].

The goal of the research reported here was to provide information on the suitability of selected soybean, canola, and corn-based products as ingredients in all-plant diets for Florida pompano, a promising aquaculture candidate in the United States. Interest in pompano aquaculture has persisted for over 50 years, but only recently has knowledge of the nutritional needs of pompano begun to increase [27]–[33]. Development of effective all-plant diets for Florida pompano could increase the profitability of pompano aquaculture, facilitate increased commercial pompano production, and assist the creation of sustainable aquaculture methods for related marine fish species. This paper presents information that can be used by nutritionists and feed formulators to optimize the use of these plant products in compounded pompano diets.

## Materials and Methods

### Ethics statement

Procedures for animal care and handling were approved by the Institutional Animal Care and Use Committee of Louisiana State University (protocol number 08-070).

### Experiment 1: Utilization of all-plant, soybean-based diets by Florida pompano

#### 1A. Diet preparation

Six isonitrogenous and isocaloric diets were formulated to match or exceed the dietary essential amino acid levels in whole-body pompano (Tables 1 and 2). Five experimental diets were formulated to contain 0, 20, 25, 30, and 35 percent soybean meal (SBM), with the remaining protein provided by soybean protein concentrate (SPC) at levels of 59, 46, 43, 39 and 36 percent, respectively. These diets were tested with a control diet that contained 30 percent SBM and 30 percent SPC in combination with 10 percent menhaden fish meal. All diets were supplemented with 0.5 percent taurine [6], [12], [14], [15], [34] and 1,000 FTU of fungal phytase (Natuphos™, BASF Corp., Geismar, Louisiana) per kilogram diet. Diets were prepared by grinding ingredients to less than 0.6-mm particle size with a Wiley mill (Thomas Scientific, Swedesboro, New Jersey). Dry ingredients were mixed for 60 minutes in a twin-shell laboratory mixer (Patterson-Kelley Co., East Stroudsburg, Pennsylvania), then moved to a Hobart food mixer (Rapids Machinery, Troy, Ohio) where oil and water were added and the wet mash was mixed to a dough-like consistency. Diets were pelletized through a 3-mm die using a pasta maker (Ronco Inventions, Chatsworth, California). Pellets were air-dried overnight, stored in sealed bags and refrigerated (4°C) until used.

**Table 1:** Composition of the experimental diets as fed (g/kg).

Ingredient	Control	M0/C59	M20/C46	M25/C43	M30/C39	M35/C36
Soybean meal	300.0		200.0	250.0	300.0	350
Soy protein concentrate	300.0	594.9	460.8	427.2	393.7	360.1
Fish meal	100.0					
Wheat flour	128.3	227.3	159.8	143.0	126.1	109.2
Fish oil	94.1	99.8	100.7	100.9	101.2	101.4
Corn gluten meal	20.0	20.0	20.0	20.0	20.0	20.0
CaH_4_(PO_4_)_2_·H_2_O	16.0	16.0	16.0	16.0	16.0	16.0
CMC^1^	10.0	10.0	10.0	10.0	10.0	10.0
Methionine	7.3	7.6	8.3	8.5	8.7	8.9
Taurine	5.0	5.0	5.0	5.0	5.0	5.0
Vitamin mix^2^	5.0	5.0	5.0	5.0	5.0	5.0
Mineral mix^3^	2.5	2.5	2.5	2.5	2.5	2.5
Soy lecithin	16.0	16.0	16.0	16.0	16.0	16.0
Choline chloride	0.9	0.9	0.9	0.9	0.9	0.9
Stay-C^4^	0.6	0.6	0.6	0.6	0.6	0.6
Phytase	0.2	0.2	0.2	0.2	0.2	0.2
Ethoxyquin	0.4	0.4	0.4	0.4	0.4	0.4
Chemical composition (g/kg, as fed)						
Dry matter	917	915	920	912	926	932
Crude protein	399	388	400	395	415	423
Lipid	112	98	94	89	87	103
Ash	76	56	60	59	65	70
Fiber	39	31	37	46	37	37
NFE^5^	290	342	330	323	323	299
Gross energy (kcal/g)	4.5	4.4	4.6	4.6	4.7	4.7

M = soybean meal; C = soy protein concentrate.

1 Carboxymethylcellulose, sodium salt.

2 Per kg diet: vitamin A, 6000 IU; vitamin D, 1000 IU; vitamin E, 0.1 g; biotin, 0.2 g; folic acid, 9 mg; niacin, 0.2 g; pantothenic acid, 0.1 g; vitamin B-6, 25 mg; riboflavin, 40 mg; thiamin, 40 mg; vitamin B-12, 20 mg.

3 Per kg diet: iron, 0.1 g; manganese, 25 mg; copper, 10 mg; zinc, 0.1 g; iodine, 4.5 mg; cobalt, 50 mcg; selenium, 0.5 mg.

4 Stay-C stabilized vitamin C (L-ascorbyl-2-polyphosphate), 35% ascorbic acid activity.

5 Nitrogen-free extract = dry matter−(crude protein+lipid+ash+fiber).

**Table 2:** Quantities of dietary essential amino acids, expressed in proportion to lysine^1^, in the whole-body of pompano and in the experimental diets.

Amino acid	WB^2^	Control	M0/C59	M20/C46	M25/C43	M30/C39	M35/C36
Arginine	0.60	0.69	0.76	0.83	0.76	0.71	0.72
Histidine	0.22	0.30	0.30	0.36	0.31	0.29	0.29
Isoleucine	0.56	0.72	0.79	0.87	0.79	0.74	0.75
Leucine	0.92	1.12	1.23	1.35	1.21	1.15	1.17
Lysine	1.00	1.00	1.00	1.00	1.00	1.00	1.00
Methionine	0.30	0.27	0.25	0.29	0.26	0.23	0.24
Phenylalanine	0.39	0.57	0.63	0.69	0.63	0.59	0.60
Threonine	0.52	0.52	0.54	0.61	0.55	0.51	0.52
Valine	0.74	0.85	0.93	1.02	0.92	0.86	0.88

M = soybean meal; C = soy protein concentrate.

1
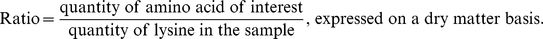

2 Whole-body pompano.

#### 1B. Experimental fish and culture system

Wild pompano were obtained with a beach seine from the surf zone of Grand Isle and Holly Beach, Louisiana. Fish were transported to the Louisiana State University Agricultural Center Aquaculture Research Station, Baton Rouge, Louisiana and subjected to a 35-day quarantine period during which they were treated to eliminate external and internal parasites (Appendix S1). The fish were then stocked into a recirculation culture system and given a one-week acclimation period prior to the start of the experiment. The recirculation system consisted of 18, circular, 227-L fiberglass tanks; a 0.3-mm mechanical particle filter (CeTus, Evolution Aqua Ltd, United Kingdom); a biological bead filter (Sweetwater, Aquatic Ecosystems Inc., Apopka, Florida); two, 120-watt, ultraviolet light sterilizers (Emperor Aquatics, Pottstown, Pennsylvania); and a corona-discharge ozone generator (CD-7, Del Ozone, San Luis Obispo, California). Salinity was maintained with synthetic sea salt (Crystal Sea, Marine Enterprises International, Baltimore, Maryland). Aeration was supplied to each tank with an airstone attached to a regenerative blower. Weekly water exchanges of approximately five percent of system volume were performed with freshly made sea water. Mean water quality parameters ± SD were: temperature, 30.1±0.9°C; salinity, 10.2±0.8 g/L; alkalinity, 97±40 mg/L; total ammonia-nitrogen, 0.3±0.4 mg/L; and nitrite-nitrogen, 0.5±0.5 mg/L. Twenty pompano (mean weight of 7.0 g per fish) were stocked in each tank, with each diet assigned to three tanks. Fish were fed to apparent satiation twice daily for ten weeks. Apparent satiation was achieved by allowing each tank to consume as much feed as possible in two, two-minute periods. Fish in each tank were batch weighed bi-weekly. Before weighing, fish were anesthetized with MS-222 (150 mg/L) and allowed to recover before being returned to the culture tank. Prior to the experiment, 15 fish were euthanized and stored frozen (−20°C) for proximate analysis. This was repeated at the conclusion of the 10-week feeding trial with three fish collected from each tank.

#### 1C. Chemical and statistical analysis

Proximate analysis of pompano tissue (whole-body) and the experimental diets was conducted by standard methods [35]. Dry matter content of diets was determined by 24-hr oven drying at 100°C; dry matter content of pompano whole-body was determined by 48-hr freeze drying. Crude protein, lipid, and fiber contents of samples were determined by the Louisiana State University Agricultural Center Department of Agricultural Chemistry. Ash content was measured by incineration at 600°C in a muffle furnace. Gross energy was determined by bomb calorimetry (Model C5000, IKA Works Inc., Wilmington, North Carolina). Nitrogen-free extract (NFE) was calculated as: NFE (%) = dry matter−(crude protein+lipid+ash+fiber). Amino acid concentrations were determined with high performance liquid chromatography after hydrolysis (1100 Series, Agilent Technologies, Wilmington, Delaware).

Data were subjected to one-way analysis of variance (ANOVA) using the mixed procedure of SAS version 9.1 (SAS, Cary, North Carolina). Prior to ANOVA, percentage data were arcsine-transformed. All hypotheses were tested at a significance level of α = 0.05. If a significant difference (*P*<0.05) was found, differences among means were identified with the Tukey-Kramer multiple range test.

### Experiment 2: Digestibility of canola meal, corn gluten meal, and distillers dried grains and solubles in compounded diets for Florida pompano

#### 2A. Diet preparation

A reference diet and three test diets containing 30 percent test ingredient — canola meal (CM), corn gluten meal (CGM), or distillers dried grains with solubles (DDGS) — and 70 percent reference diet were formulated (Table 3). Chromic oxide was used as an inert marker (10 g/kg diet). Diets were prepared as described in section 1A, after which the chemical compositions of the finished diets and the test ingredients were determined by analysis (Table 4).

**Table 3:** Ingredient composition of the reference diet and test diets as fed (g/kg).

Ingredient	Reference diet	Test diet
Fish meal, menhaden	379.4	265.6
Soybean meal (49% CP)	250.0	175.0
Soy protein concentrate	100.0	70.0
Wheat middlings	153.3	107.3
Oil, menhaden	65.0	45.5
Soy lecithin	10.0	7.0
CMC^1^	20.0	14.0
Vitamin mix^2^	5.0	3.5
Mineral mix^3^	2.5	1.8
Stay-C^4^	0.6	0.4
Chromic oxide	10.0	7.0
Ethoxyquin	0.2	0.1
Choline chloride	4.0	2.8
Test ingredient^5^		300.0

1 Carboxymethylcellulose, sodium salt.

2 Per kg diet: vitamin A, 6000 IU; vitamin D, 1000 IU; vitamin E, 0.1 g; biotin, 0.2 g; folic acid, 9 mg; niacin, 0.2 g; pantothenic acid, 0.1 g; vitamin B-6, 25 mg; riboflavin, 40 mg; thiamin, 40 mg; vitamin B-12, 20 mg.

3 Per kg diet: iron, 0.1 g; manganese, 25 mg; copper, 10 mg; zinc, 0.1 g; iodine, 4.5 mg; cobalt, 50 mcg; selenium, 0.5 mg.

4 Stay-C stabilized vitamin C (L-ascorbyl-2-polyphosphate), 35% ascorbic acid activity.

5 Canola meal, corn gluten meal, or distillers dried grains with solubles.

**Table 4:** Chemical composition of the diets and test ingredients as fed (%).

	Diet				Ingredient		
	Reference	CM^1^	CGM^2^	DDGS^3^	CM	CGM	DDGS
Dry matter	92.70	91.98	91.39	91.94	89.2	89.4	87.6
Crude protein	46.91	43.95	51.61	39.78	36.3	61.9	25.8
Lipid	11.02	8.48	8.54	11.50	4.9	4.2	13.9
Ash	11.80	10.61	8.63	9.64	7.3	1.1	3.7
Chromic oxide	1.08	0.73	0.82	0.85			
GE (kcal/g)^4^	4.65	4.48	4.75	4.72	4.2	5.0	4.5
Fiber					9.7	0.3	5.8
NFE^5^					31.0	21.9	38.4

Values determined by analysis.

1 Canola meal.

2 Corn gluten meal.

3 Distillers dried grains with solubles.

4 Gross energy.

5 Nitrogen-free extract = dry matter−(crude protein+lipid+ash+fiber).

#### 2B. Experimental fish and culture system

Wild Florida pompano were collected and treated as described in section 1B. The fish were then stocked into 12 tanks of the recirculation culture system described in section 1B and grown to a mean individual weight of at least 80 g before digestibility trials were initiated. During the grow-out period fish were fed a commercial marine starter diet (Aquaxcel, Cargill, Franklinton, Louisiana). Weekly water exchanges of approximately five percent of system volume were performed with freshly made sea water. Mean water quality parameters ± SD were: temperature, 26.0±0.7°C; salinity, 9.9±0.6 g/L; alkalinity, 91±15 mg/L; total ammonia-nitrogen, 0.3±0.2 mg/L; and nitrite-nitrogen, 0.4±0.2 mg/L.

Twenty-five pompano (mean weight of 83 g per fish) were stocked into each tank, with each diet assigned to three tanks. Fish were fed once daily and were acclimated to diets for six days prior to fecal collection. Fecal material was collected weekly for five weeks. On collection days, fish were fed to apparent satiation. To prevent leaching losses [36], fecal material was manually stripped 3.5 to 4 hr after feeding by applying pressure to the abdomen [37], [38], [39]. Prior to stripping, fish were anesthetized with MS-222 (150 mg/L) and allowed to recover before being returned to the culture tank. Fecal samples from individual fish were pooled, by tank, and stored frozen at −20°C. Samples were freeze-dried for a minimum of 48-hr prior to chemical analysis.

#### 2C. Chemical and statistical analysis

Proximate analysis, energy content, and amino acid analysis of diets and fecal samples were determined as described in section 1C. Chromium content of diets and feces was determined with inductively coupled plasma spectrometry by the Louisiana State University Agricultural Center Department of Agricultural Chemistry.

Apparent digestibility coefficients (ADCs) for each nutrient of interest were calculated in two steps. First, ADCs for a given nutrient in both the reference diet and test diets were determined with equation 1 [40]:
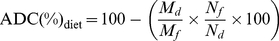
(1)where *diet* is reference diet or test diet; *M_d_* and *M_f_* are percentages of chromic oxide marker in the diet and feces, respectively; and *N_f_* and *N_d_* are percentages of the nutrient of interest (e.g., crude protein, energy, or a particular amino acid) in the feces and diet, respectively. Equation 2 [41] was then used to calculate the corrected ADC for each nutrient of interest in each ingredient of interest based upon the 70∶30 ratio of reference diet-to-test ingredient in each of the test diets:
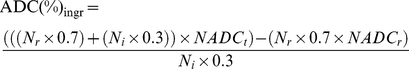
(2)where *ingr* is the test ingredient of interest; *N_r_* and *N_i_* are percentages of the nutrient of interest (e.g., crude protein, energy, or a particular amino acid) in the reference diet and test ingredient, respectively; *NADC_t_* is the ADC of the nutrient of interest in the test diet, determined with equation 1; and *NADC_r_* is the ADC of the nutrient of interest in the reference diet, determined with equation 1.

ADCs were subjected to one-way ANOVA as described in section 1C. If indicated, significant (*P*<0.05) differences among means were identified with the Tukey-Kramer multiple range test. ADCs of selected ingredients also were subjected to regression analysis (SAS version 9.1).

## Results

### Experiment 1: Utilization of all-plant, soybean-based diets by Florida pompano

Daily consumption of the M0/C59 diet was observed to be less than that of the other diets during the feeding trial. Pompano fed the M0/C59 diet had significantly lower weight gain, specific growth rate (SGR) and feed intake (FI), and significantly higher (poorer) feed conversion ratio (FCR), than fish fed the other diets (Table 5). Protein efficiency ratio (PER) of M0/C59-fed fish did not differ (*P*>0.05) from PER of fish fed M30/C39, but was lower than PER of fish fed the other diets (Table 5). Significant differences in survival were identified, but there was no apparent relationship related to dietary treatment (Table 5). Fish fed the control diet had significantly higher weight gain than fish fed M0/C59, M20/C46, or M35/C36, but did not differ (*P*>0.05) in weight gain from fish fed M25/C43 or M30/C39 (Table 5). With the exception of the control diet which contained fish meal, no significant differences in weight gain, FCR, SGR, PER, FI, or survival were observed among pompano in the treatment groups that received diets containing both soybean meal (20–35 percent) and soy protein concentrate (36–46 percent) as protein sources (Table 5).

**Table 5:** Growth parameters of Florida pompano fed the experimental diets for 10 weeks.

Diet	Weight gain^1^	FCR^2^	SGR^3^	PER^4^	FI^5^	Survival (%)
Control	1075±71^a^	1.56±0.04^a^	3.52±0.09^a^	1.60±0.04^a^	1.60±0.12^a^	98±2^ab^
M0/C59	410±42^c^	2.18±0.15^b^	2.32±0.11^c^	1.20±0.08^b^	0.80±0.07^c^	82±6^b^
M20/C46	758±50^b^	1.76±0.06^a^	3.06±0.08^b^	1.43±0.05^a^	1.33±0.03^ab^	95±5^ab^
M25/C43	875±55^ab^	1.61±0.01^a^	3.25±0.08^ab^	1.58±0.01^a^	1.42±0.10^ab^	100^a^
M30/C39	878±29^ab^	1.69±0.01^a^	3.26±0.04^ab^	1.42±0.01^ab^	1.50±0.03^ab^	100^a^
M35/C36	793±73^b^	1.64±0.05^a^	3.12±0.12^ab^	1.44±0.05^a^	1.28±0.08^b^	93±4^ab^

Mean values ± SE (n = 3), values in each column with different letters are significantly different (*P*<0.05).

M = soybean meal; C = soy protein concentrate.

1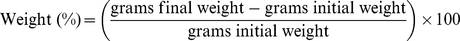.

2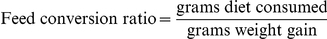.

3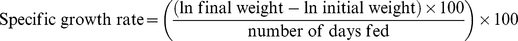.

4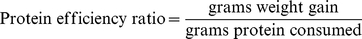.

5 Feed intake, g/fish/day.

Whole-body of pompano fed the M0/C59 diet contained significantly less crude protein per gram (dry weight) than whole-body of fish fed the control diet, but did not differ (*P*>0.05) in protein content from whole body of fish fed the other diets (Table 6). Moisture content of fish fed M0/C59 was higher (*P*<0.05) than that of fish fed the control diet or the M30/C39 diet, but not different (*P*>0.05) from the moisture content of fish fed M20/C46, M25/C43, or M35/C36. No trend in lipid, ash, or energy content of fish whole-body was apparent among treatment groups, indicating no observed effect of soy-product inclusion levels on these components of body composition.

**Table 6:** Whole-body composition of Florida pompano fed the experimental diets.

Diet	Moisture (g/100 g)	Crude protein^1^ (g/100 g)	Lipid (g/100 g)	Ash (g/100 g)	GE^2^ (kcal/g)
Control	69.7±0.4^b^	17.3±<0.1^a^	9.0±0.4^ab^	2.9±<0.1^b^	5.8±0.8^ab^
M0/C59	73.5±0.9^a^	16.3±0.3^b^	5.2±1.2^b^	3.3±<0.1^a^	5.3±1.8^b^
M20/C46	69.9±0.6^ab^	17.0±0.2^ab^	8.9±0.4^ab^	2.9±0.1^b^	5.8±0.3^ab^
M25/C43	71.5±1.1^ab^	16.5±0.2^ab^	7.5±1.3^ab^	3.0±0.1^ab^	5.6±1.6^ab^
M30/C39	68.7±1.1^b^	16.8±0.1^ab^	9.8±0.9^a^	2.8±0.1^b^	5.9±1.2^a^
M35/C36	70.9±0.3^ab^	17.0±0.3^ab^	7.8±0.1^ab^	3.0±0.1^ab^	5.7±0.5^ab^

Mean values ± SE (n = 3), values in each column with different letters are significantly different (*P*<0.05). Initial body composition prior to treatment (g/100 g): moisture, 68.9; crude protein, 14.9; lipid, 10.5; ash, 3.0; GE, 6.1.

M = soybean meal; C = soy protein concentrate.

1 Crude protein = percentage nitrogen×6.25.

2 Gross energy as determined by bomb calorimetry.

### Experiment 2: Digestibility of canola meal, corn gluten meal, and distillers dried grains and solubles in compounded diets for Florida pompano

Apparent crude protein digestibility (ACPD) ranged from 20.6 percent for DDGS to 57.2 percent for CGM (Table 7). Crude protein digestibility of CGM was significantly higher than that of DDGS, but neither CGM nor DDGS differed significantly from CM in protein digestibility. Apparent energy digestibility (AED) ranged from 21.3 percent to 57.1 percent (Table 7). AED of CGM was significantly higher than AED of CM or DDGS. DDGS also had higher (*P*<0.05) energy digestibility than CM.

**Table 7:** Apparent digestibility coefficients of crude protein (ACPD) and energy (AED) in ingredients fed to Florida pompano.

Ingredient	ACPD	AED
Canola meal	38.6±4.3^ab^	21.3±2.1^c^
Corn gluten meal	57.2±2.4^a^	57.1±2.5^a^
Distillers dried grains with solubles	20.6±7.0^b^	30.7±1.4^b^

Mean values ± SE (n = 3), values in each column with different letters are significantly different (*P*<0.05).

Apparent amino acid availability (AAAA) of leucine and tyrosine was significantly higher in CGM than in CM or DDGS (Table 8). AAAA of alanine and proline was significantly higher in CGM than in CM or DDGS, while availability of these amino acids in CM was significantly below their availability in DDGS. AAAA of aspartic acid and glycine in CGM was significantly higher than in DDGS but not different (*P*>0.05) than availability of these amino acids in CM. No other significant differences were detected in AAAA among ingredients. Statistically significant differences in mean AAAA of amino acid groups also were not identified among ingredients (Table 9).

**Table 8:** Apparent availability coefficients of dietary essential and non-essential amino acids (AA) in ingredients fed to Florida pompano.

	CM^1^	CGM^2^	DDGS^3^
Essential AA			
Arginine	53.8±5.9	68.5±10.8	35.0±19.7
Histidine	46.9±8.2	58.7±6.1	30.0±19.3
Isoleucine	50.4±8.0	62.5±9.8	40.9±10.9
Leucine	46.8±3.5^b^	70.8±5.2^a^	55.6±5.1^b^
Lysine	48.4±3.1	47.9±14.4	50.4±41.2
Methionine	91.9±0.4	84.9±3.8	91.5±4.6
Phenylalanine	54.2±8.2	70.9±6.6	55.5±7.9
Threonine	44.6±8.8	56.9±10.5	37.6±13.1
Valine	48.1±6.1	64.7±8.2	50.4±7.9
Non-essential AA			
Alanine	32.7±3.9^c^	68.3±4.7^a^	44.9±5.0^b^
Aspartic acid	17.5±8.2^ab^	42.3±10.4^a^	11.8±12.5^b^
Cysteine	30.3±15.6	42.5±19.2	23.0±20.8
Glutamic acid	49.8±6.0	61.1±5.3	42.2±10.2
Glycine	17.7±6.9^ab^	34.9±13.4^a^	8.3±8.5^b^
Proline	32.1±4.0^c^	61.0±5.1^a^	46.4±5.3^b^
Serine	45.4±20.9	81.1±2.3	63.1±20.5
Tyrosine	44.0±0.6^b^	77.5±2.1^a^	49.0±2.8^b^
Grand mean of AA	44.4±16.7	62.0±14.0	43.3±19.5

Mean values ± SD (n = 3), values in each row with different letters are significantly different (*P*<0.05).

1 Canola meal.

2 Corn gluten meal.

3 Distillers dried grains with solubles.

**Table 9:** Apparent availability of amino acids, by type, in ingredients fed to Florida pompano.

Group^1^	CM^2^	CGM^3^	DDGS^4^
Acidic	33.6±22.9	51.7±13.3	27.0±21.5
Basic	49.7±3.6	58.4±10.3	38.5±10.6
Non-polar	50.9±20.0	69.0±8.0	55.0±17.0
Uncharged polar	36.4±12.2	58.6±20.5	36.2±21.5

Values are mean percentage availability ± SD.

1 Acidic: aspartic acid, glutamic acid; Basic: arginine, histidine, lysine; Non-polar: alanine, isoleucine, leucine, methionine, phenylalanine, proline, valine; Uncharged polar: cysteine, glycine, serine, threonine, tyrosine.

2 Canola meal.

3 Corn gluten meal.

4 Distillers dried grains with solubles.

## Discussion

### Experiment 1: Utilization of all-plant, soybean-based diets by Florida pompano

Results of the current study suggest that replacement of fish meal with mixtures of SBM and SPC is feasible for Florida pompano, but factors other than the amino acid profile of these ingredients affect fish performance at different levels of soybean-product inclusion. The significantly lower feed intake of fish fed M0/C59 was a primary cause of the poor weight gain of fish in this treatment group due to reduced nutrient intake relative to fish fed the other diets (Table 5). It is possible that one of the factors affecting feed intake is the attractiveness or palatability of soybean products. Feed intake data suggest that a diet (M0/C59) composed primarily of SPC (59 percent), wheat flour, fish oil, and corn gluten meal was not as attractive and/or palatable as diets of similar composition and nutritional value that contained 20–35 percent SBM and reduced quantities of SPC (36–46 percent of diet). Why this would be the case is not readily apparent. However, the nutritional equivalence — i.e., amino acid (Table 2) and energy content (Table 1) — among diets suggests that nutrient deficiencies were unlikely to be the cause. There is no evidence from previous research conducted in this laboratory, or from the literature, to suggest that SBM in prepared diets is attractive to pompano, but it is possible that SPC could be more unattractive than SBM, such that replacement of 13 percent SPC in the M0/C59 diet with SBM in the M20/C46 diet significantly increased feed intake to a level not different (*P*>0.05) from that of fish fed the control diet (Table 5). This effect appears to be limited, however, since feed intake and weight gain of pompano fed M35/C36, which contained the largest amount of soy products (710 g/kg diet), also was significantly below that of fish fed the control diet.

Weight gain, FCR, SGR, and PER did not differ significantly among Florida pompano fed the control diet, M25/C43 or M30/C39, indicating that a 40 percent protein, fish-meal-free (FMF) diet containing 680–690 g/kg (68–69 percent) of SPC and SBM produced growth performance of pompano equivalent to a similar diet (600 g/kg SPC and SBM) containing 10 percent (100 g/kg) menhaden fish meal.

Studies with other species have shown reduced growth of fish fed diets that contained SPC as a major protein source. Kissil et al. [42] reported that increased levels of SPC and phytic acid in diets for gilthead seabream caused reduced feed intake and weight gain due to low diet palatability. Deng et al. [9] improved the palatability of soy-based diets for Japanese flounder by incorporating 0.5 percent taurine as a feeding stimulant, and reduced phytic acid content by adding phytase at a concentration of 750 FTU/kg diet. Nonetheless, replacement of 87.5 percent and 100 percent of fish meal with SPC resulted in decreased growth of Japanese flounder due to reduced diet acceptability.

Zhao et al. [43] completely replaced fish meal with SPC in Nile tilapia diets by increasing feeding frequency. Nile tilapia fed a FMF soybean-based diet six times per day exhibited feed intake and weight gain not different from that of fish fed a fish-meal based diet twice per day. Walker et al. [44] reported no negative effects of SPC inclusion level on growth or feed intake of Atlantic cod fed FMF diets. However, hydrolyzed fish protein concentrate and blood meal, which are likely feeding stimulants, also were included in all diets. Burr et al. [45] replaced up to 82 percent of fish meal in diets for 20-g rainbow trout with a soy-based protein blend, with no negative effects on growth. A similar plant-protein blend depressed growth of 6-g Atlantic salmon when used to replace 50 percent of dietary fish meal, but growth of late-stage juvenile Atlantic salmon (30-g or larger) was unaffected by complete replacement of fish meal with a blend of corn protein concentrate, SPC and supplemental amino acids [45].

Soybean products are among the most promising replacements for fish meal in aquafeeds. However, to be effective, FMF diets must be consumed in quantities sufficient to support rapid fish growth and cost-efficient production. Development of nutritious, palatable, FMF diets for Florida pompano, and other fishes, will require the continued identification and testing of new alternatives to fish meal.

### Experiment 2: Digestibility of canola meal, corn gluten meal, and distillers dried grains and solubles in compounded diets for Florida pompano

Results indicated that protein digestibility of CGM was significantly higher than that of DDGS, but not significantly different from protein digestibility of CM, suggesting one or more negative attributes of DDGS that could reduce its value as a protein source for pompano diets. DDGS contains 9–11 percent fiber but no antinutritional factors other than phytic acid which, as the primary storage form of phosphorus in plants, is ubiquitous in feedstuffs of plant origin [2]. Phytic acid can bind to protein, amino acids, and minerals in the diet causing decreased availability [46]. CM contains 12–13 percent cellulose fiber, 1–2 percent indigestible oligosaccharides in dehulled meal (higher in common meal) and, in addition to phytic acid, a number of antinutritional factors (i.e., tannins, sinapine, glucosinolates) that can reduce the nutritional value of CM by negatively affecting digestion and nutrient absorption [19]. However, many of these antinutritional compounds are located in the canola seed hull and concentrations are greatly reduced in dehulled CM of the type used in this study. In comparison, CGM contains less than three percent fiber and no antinutritional factors [24].

**Figure 1:**
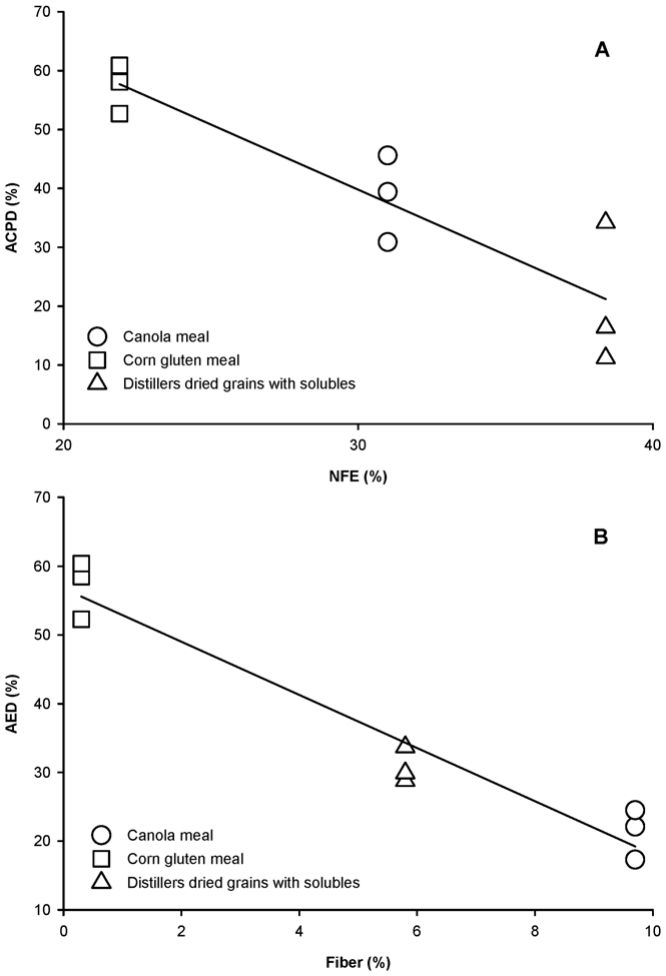
Influence of carbohydrate and fiber on digestibility. Simple linear regression demonstrates the negative relationship between apparent crude protein digestibility (ACPD) and nitrogen-free extract (NFE) (plot A; *r* = −0.91), and apparent energy digestibility (AED) and fiber (plot B; *r* = −0.97) among the three ingredients tested. NFE is primarily starches and other carbohydrates; fiber is mostly cellulose. As concentrations of NFE or fiber increase, Florida pompano digest protein and energy in the feedstuffs less efficiently.

High quantities of indigestible starch, fiber, and antinutritional factors in a feedstuff can be expected to negatively affect protein and energy digestibility. Figure 1 confirms the inverse relationship between the starch (NFE) and fiber content of CM, CGM and DDGS, and the protein and energy digestibility of these ingredients. ACPD was negatively correlated (*r* = −0.91) with NFE content (i.e., CGM>CM>DDGS; Figure 1A), while AED was negatively correlated (*r* = −0.97) with fiber content (i.e., CGM>DDGS>CM; Figure 1B).

Plant products incorporated into feeds as protein sources also contain carbohydrates and fiber. Many fishes have limited ability to digest carbohydrates and no ability to digest fiber. In the current study, increased dietary NFE was associated with decreased ACPD, supporting the contention that dietary carbohydrate level affects protein digestion. The mechanism that produces this effect is thought to be primarily physical. Nutrient absorption is maximized when there is sufficient gut-retention time to allow full digestion of foods and absorption of the nutrients released. Nutrients can be absorbed only if they come in contact with appropriate cells lining the gastrointestinal tract. As in most carnivorous fishes, the gastrointestinal tract of Florida pompano is short — less than one body length — which limits the gut-retention time to approximately three hours (personal observation). Carbohydrates, if present in sufficient quantity, can cause diarrhea in some fishes, further decreasing gut-retention time and reducing the time available for nutrient absorption. Carbohydrates also can affect digestibility by physically preventing digestive enzymes from contacting substrates in the intestinal lumen, thereby slowing the rate of food digestion and nutrient uptake. Fiber can have a similar physical effect on food digestion through interference with nutrient absorption and gut-retention time [5]. However, the negative correlation of AED with fiber content in Figure 1B is a direct effect of fiber indigestibility. Fiber constitutes a portion of the gross energy of most diets but has no digestible energy value to monogastric species, including most fishes. Because the energy in fiber is unavailable, the AED of a diet typically decreases as fiber content increases.

Mean ACPD and AED coefficients reported in this study were lower than those previously reported for Florida pompano and some other carnivorous fishes [20], [29], [33], [47]–[60] (Table S1). Riche and Williams [33] reported protein and energy digestibility of CGM to be 82–83 percent and 77 percent, respectively, for Florida pompano cultured in brackish water (3 g/L or 28 g/L salinity). Williams [29] reported protein and energy digestibility of DDGS to be 54–60 percent and 63–66 percent, respectively, for Florida pompano. Despite differences in the numerical values of coefficients determined in this study and those of Riche and Williams [33], and Williams [29], results of these three studies trend toward CGM having greater apparent digestibility for Florida pompano than DDGS.

Burel and Kaushik [19] reported that protein digestibility of CM is greater than 80 percent for fishes; however, energy digestibility can vary widely, from 21–83 percent. CM appears to be well digested by some fish but was not digested well by pompano in this study. Protein digestibility of CM, however, was similar to that of CGM and higher than protein digestibility of DDGS.

It is assumed in digestibility measurements that diet digestibility is the sum of the digestibility of individual diet ingredients. Thus, the ADC of a given nutrient in a diet should be calculable by summing the proportional ADCs for that nutrient in each of the individual ingredients composing the diet [61]. Based on the protein and energy ADCs of soybean meal [32], soy protein concentrate [62] and fish meal [62] established in previous feeding trials in this laboratory, the ACPD of the reference diet used in this study should have been approximately 90 percent. However, ACPD and AED of the reference diet were calculated to be 67 percent and 55 percent, respectively, indicating that ADCs for protein and energy in the reference diet ingredients were not additive in the current study. ADCs also are assumed to be constant, regardless of test ingredient inclusion level, and to be unaffected by the inclusion levels of other ingredients. However, in practice, interactions among diet ingredients do occur and the effects of such interactions on diet digestibility and nutrient availability can be difficult to predict. In the current study, apparent protein and energy digestibility of fish meal, soybean meal and soy protein concentrate were not as high, when combined in the proportions present in the reference diet, as has been shown possible for these ingredients in other studies with Florida pompano in this laboratory.

There are numerous factors that can affect digestibility measurements, including diet composition, feed intake, fish size, fecal collection method and diet processing, among others [2], [39], [47]. It can be difficult to determine the reasons for variations in nutrient digestibility measurements among laboratories, or even within a laboratory during a period of time, although lack of methods standardization is a factor. For example, the 25 percentage-point difference between the protein digestibility of CGM obtained in the current study and that reported by Riche and Williams [33] could be associated with the use of very dissimilar reference diets in the two studies, since culture methods were similar.

**Figure 2:**
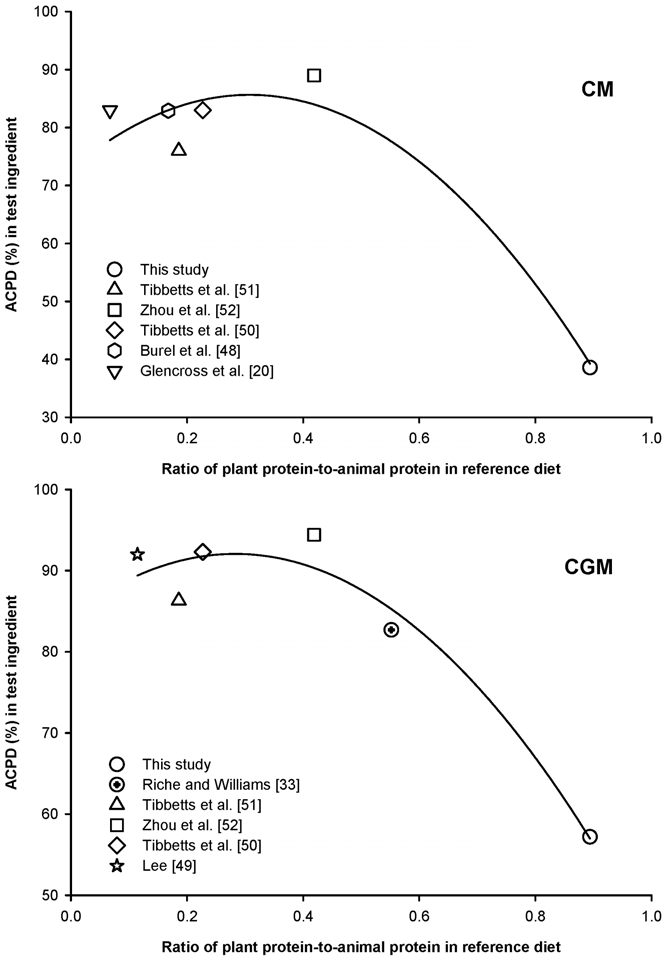
Reference diet composition affects digestibility measurements. The quantity of nitrogen-free extract (carbohydrates) and fiber in a prepared diet often increases with the amount of plant products in the formulation. Diet digestibility can be negatively affected as plant products replace ingredients of animal origin and the ratio of plant products to animal products increases. Figure 2 illustrates the relationship between the calculated apparent crude protein digestibility (ACPD) of canola meal (CM) and corn gluten meal (CGM) in studies with Florida pompano and other carnivorous marine fishes (Table S1). Curves represent a second order polynomial. A high ratio of plant protein to animal protein (i.e., higher quantity of plant products) in the reference diet is associated with reduced ACPD in the test ingredient. This suggests that reference diet composition can affect digestibility measurements and should be considered when evaluating ingredients to provide the most useful results. Accuracy of calculated digestibility coefficients may be improved by using a reference diet with composition similar to the practical formulation in which the test ingredient will be used.

There is no universally accepted reference diet for digestibility trials. Reference diets vary in composition depending on many factors, including the experimental objectives of a research project. The reference diet used in this study contained a higher percentage of protein from plant products and a lower percentage of protein from animal products than reference diets previously used in digestibility studies with Florida pompano and some other marine species (Table S1). Figure 2 illustrates the relationship between dietary protein source (animal and plant), among reference diets used in several published digestibility studies with Florida pompano and other marine species (Table S1), and calculated protein digestibility of CM and CGM. Similar information for DDGS was not available in the literature.

ACPD of CM and CGM in this study — 39 percent and 57 percent, respectively — were considerably lower than ACPD coefficients previously reported for these ingredients fed to Florida pompano and other marine species (Figure 2). Reference diets reported by Glencross et al. [20], Riche and Williams [33], Burel et al. [48], Lee [49], Tibbetts et al. [50], [51], and Zhou et al. [52] were formulated to contain high (greater than 60 percent) concentrations of dietary protein from animal sources and therefore low levels of crude protein from plant materials. In the current study, however, animal (fish meal) protein and plant (soybean and wheat) protein sources provided similar quantities of dietary protein. This suggests that the quantity of plant-protein supplements in a reference diet may affect the apparent digestibility of protein in a test ingredient mixed with that diet in a 70/30 ratio. Thus, it is possible that reference diets containing high levels of crude protein from plant ingredients may yield lower ACPD coefficients for test ingredients combined with them than reference diets that contain high levels of animal protein products. It is not possible to prove this hypothesis with the data collected in the current study, but a relationship between reference diet composition and digestibility of protein in plant products fed to Florida pompano is indicated by the results of the current study.

The current study was conducted over a five-week period to allow adequate collection of fecal samples. The duration of the experiment also could have contributed to the low ADCs reported in the current study. Negative effects of plant products are likely magnified as exposure time is increased. ADCs only represent a snapshot of digestibility when conducted over a short period. Animals and their gut micro-flora communities can adapt to changes in diet composition to increase digestive efficiency, however chronic exposure to high levels of plant products could decrease digestive efficiency of species susceptible to antinutritional factors in plants.

It should be remembered that there is no single, “true” digestibility value for any nutrient in a feedstuff. ADCs are variable and will fluctuate with multiple environmental and physiological factors. However, ADCs determined for the same species of fish fed the same feedstuff should be relatively consistent when the animal is cultured under similar conditions.

Results of the current study, when compared with work done with Florida pompano at other laboratories, indicate that nutrient availability of feedstuffs can vary considerably among studies, even when culture conditions, fish size, and experimental methods are similar. This suggests that efforts in recent years to improve the accuracy and precision of mathematical equations for digestibility calculations [41] may address only part of the standardization problem. Information on effects of ingredient combinations and ingredient-inclusion levels on the digestibility of individual feedstuffs also is needed to produce realistic nutrient-digestibility coefficients for use in practical diet formulation.

Published data on nutrient availability in feedstuffs is not only species-specific, but also diet-specific. Digestibility/nutrient availability is a function not only of the chemical composition of a feedstuff itself, but also of the chemical and physical composition of the larger diet of which it is a part. Thus, reference diet composition may be another significant factor that researchers should consider more closely when measuring nutrient digestibility/availability in feedstuffs. Because the nutrient availability of an ingredient can vary among different diet formulations, the ingredient/chemical composition of reference diets used to generate digestibility data for practical feed formulation should mimic the composition of the production diets in which the data will be used, to ensure that nutrient availability coefficients are accurate in the intended application.

## Supporting Information

Table S1 Reported apparent crude protein digestibility (ACPD) and apparent energy digestibility (AED) of canola meal (CM), corn gluten meal (CGM), and distillers dried grains with solubles (DDGS) among various fish species.
(PDF)

Appendix S1 Quarantine protocol.
(PDF)
