# Supplementary material for: Plant Products Affect Growth and Digestive Efficiency of Cultured Florida Pompano (Trachinotus carolinus) Fed Compounded Diets
Source: PLoS One. 2012 Apr 20;7(4):e34981. doi: 10.1371/journal.pone.0034981 (PMC3335036; doi:10.1371/journal.pone.0034981)
Supplement: Table S1 — Reported apparent crude protein digestibility (ACPD) and apparent energy digestibility (AED) of canola meal (CM), corn gluten meal (CGM), and distillers dried grains with solubles (DDGS) among various fish species. (PDF) [file pone.0034981.s001.pdf]

Table S1. Reported apparent crude protein digestibility (ACPD) and apparent energy digestibility (AED) of canola meal (CM), corn gluten meal (CGM), and distillers dried grains with solubles (DDGS) among various fish species.

Ingredient	Species	ACPD	AED	Source
CM				
	Florida pompano	38.6	21.3	Current study[3]
	Atlantic cod	76.0	60.6	Tibbetts et al. [51][3]
	Atlantic salmon	74.1		Anderson et al. [53]
	Chinook salmon	84.5	64.5	Hajen et al. [54]
		79.0		NRC [55]
	Cobia	89.0	83.1	Zhou et al. [52][3]
	Haddock	83.0	60.1	Tibbetts et al. [50][3]
	Rainbow trout	90.9	76.4	Burel et al. [48]
	Red seabream	83.0	44.0	Glencross et al. [20][3]
	Silver perch	83.0	58.1	Allan et al. [56]
	Turbot	82.9	69.3	Burel et al. [48][3]
CGM				
	Florida pompano	57.2	57.1	Current study[3]
		81.9	77.4	Riche and Williams [33][1,3]
		83.4	77.4	Riche and Williams [33][2,3]
	Atlantic cod	86.3	82.7	Tibbetts et al. [51][3]
	Atlantic salmon	83.1		Anderson et al. [53]
	Cobia	94.4	94.2	Zhou et al. [52][3]
	Haddock	92.3	80.7	Tibbetts et al. [50][3]
	Rainbow trout	87.0		NRC [55]
		91.0		Halver and Hardy [57]
		97.3		Sugiura et al. [58]
		87.4	80.0	Cheng and Hardy [47]
	Rockfish	92.0	89.0	Lee [49][3]
	Silver perch	95.4	104.5	Allan et al. [56]
DDGS				
	Florida pompano	20.6	30.7	Current study
		54.0	63.5	Williams [29][1]
		60.5	66.2	Williams [29][2]
	Rainbow trout	85.0	51.0	Cho and Kaushik [59]
		72.0		Halver and Hardy [57]
		90.4		Cheng and Hardy [60]

[1]Salinity, 3 g/L.
[2]Salinity, 28 g/L.
[3]Reports used in Figure 2.
