# Supplementary material for: Plant Products Affect Growth and Digestive Efficiency of Cultured Florida Pompano (Trachinotus carolinus) Fed Compounded Diets
Source: PLoS One. 2012 Apr 20;7(4):e34981. doi: 10.1371/journal.pone.0034981 (PMC3335036; doi:10.1371/journal.pone.0034981)
Supplement: Appendix S1 — Quarantine protocol. (PDF) [file pone.0034981.s002.pdf]

Appendix S1: Quarantine protocol

Wild Florida pompano (*Trachinotus carolinus*) were collected in the spring with a beach seine at Holly Beach and Grand Isle, Louisiana. Fish greater than 1-cm length were transported to the Louisiana State University Agricultural Center Aquaculture Research Station, Baton Rouge, where they were slowly acclimated to water in the quarantine system by gradually replacing water in the fish hauler with water from the quarantine tank. Every 60 minutes, for a period of six hours, approximately one-quarter of the water in the hauling tank was replaced with water from the quarantine tank. This slow acclimation was necessary to avoid stress-related mortality. After acclimation, the fish were placed in a treatment tank and subjected to a three-hour, potassium permanganate bath (2 mg/L) to destroy external bacteria. Following treatment, the fish were sorted by size and placed in separate floating cages in the quarantine tank.

The quarantine system comprised one 10,000-L circular tank, filled to a volume of 4,500 L; water pump; biological bead filter; and a 120-watt ultraviolet-light sterilization unit. Aeration was provided through airstones attached to a regenerative blower. Salinity was maintained at 12 g/L with synthetic sea salts. Water temperature was determined by ambient air temperature.

Cutrine was added to the system at 0.25 mg Cu^{2+}/L and maintained at this concentration for 30 days to remove monogenetic trematodes and parasites. Each cage was given an initial, and thereafter weekly, one-hour, formalin bath (150 mg/L) in a separate treatment tank. Addition of Cutrine ceased after 30 days and Cu^{2+} dissipated within two days.

On day 33, the water level was reduced by half and a 24-hour praziquantal bath (2.5 mg/L) was given to remove gastric parasites and worms. The water level was then returned to normal and on day 35, fish were transported into the laboratory culture system.

During the quarantine period, fish were fed a marine starter diet (Aquaxcel, Cargill, Franklinton, Louisiana) twice daily and water exchanges of approximately five percent of system volume were performed daily.
